# NEAT1 upregulates EGCG-induced CTR1 to enhance cisplatin sensitivity in lung cancer cells

**DOI:** 10.18632/oncotarget.9712

**Published:** 2016-05-30

**Authors:** Pan Jiang, Xiaoyue Wu, Xuemin Wang, Wenbin Huang, Qing Feng

**Affiliations:** ^1^ Department of Nutrition and Food Hygiene, Key Laboratory of Toxicology, School of Public Health, Nanjing Medical University, Nanjing, China; ^2^ Beijing Research Institute for Nutritional Resources, Beijing, China; ^3^ Department of Pathology, Affiliated Nanjing First Hospital of Nanjing Medical University, Nanjing, China

**Keywords:** cisplatin, lung cancer, hsa-mir-98-5p, CTR1, NEAT1

## Abstract

Platinum-based drugs are the firstline of treatment for non-small cell lung cancer (NSCLC), but resistance to these drugs is a major obstacle to effective chemotherapy. Our previous study revealed that the green tea polyphenol, EGCG, induced cisplatin transporter CTR1 (copper transporter 1) and enhanced cisplatin sensitivity in ovarian cancer. In this study, we found that EGCG upregulated CTR1 and increased platinum accumulation in NSCLC (A549, H460 and H1299) cells, cDDP-resistant A549 cells and a nude mouse xenograft model. Cisplatin-induced inhibition of cell growth was enhanced by EGCG treatment *in vitro* and *in vivo*. MicroRNA hsa-mir-98-5p appears to suppress CTR1 gene expression, while long non-coding RNA (lncRNA) nuclear enriched abundant transcript 1 (NEAT1) appears to enhance it. Bioinformatics analysis showed that hsa-mir-98-5p has specific complementary binding sites for NEAT1. In addition, hsa-mir-98-5p was predicted to be a putative CTR1 target. NEAT1 may act as a competing endogenous lncRNA to upregulate EGCG-induced CTR1 by sponging hsa-mir-98-5p in NSCLC. Our findings reveal a novel mechanism how NEAT1 upregulates EGCG-induced CTR1 and enhances cisplatin sensitivity *in vitro* and *in vivo*, and suggest EGCG could serve as an effective adjuvant chemotherapeutic in lung cancer treatment.

## INTRODUCTION

Non-small cell lung cancer (NSCLC) is one of the leading causes of cancer death worldwide [1–2]. Platinum-based chemotherapy, such as cisplatin (cDDP), is a conventional treatment for most advanced NSCLC patients [3], but resistance to their therapeutics is a major obstacle. cDDP resistance mechanisms are complex, and include decreased drug absorption and increased drug loss [4]. Platinum drug transportation (import and export) and retention in tumor cells are reportedly crucial factors in treatment efficacy [4–5]. Platinum-based drug efficiency is highly impacted by their transport system and can be improved by modulating this system [6].

Copper transporter 1 (CTR1, or hCtr1 encoded by *SLC31A1*), a copper influx transporter, reportedly promotes a significant fraction of cDDP internalization in tumor cells [7–9]. cDDP resistance in cancers is associated with changes in CTR1 level, sub-cellular localization or functionality [10–11]. As the primary copper influx transporter, CTR1 controls cellular cDDP accumulation. The correlation between higher CTR1 levels and higher platinum drug uptake in tumor cells has been confirmed in a number of studies [9, 12]. CTR1 upregulation can sensitize tumor cells to platinum drugs, while CTR1 downregulation promotes resistance [9].

Our previous study showed that (−)-epigallocatechin-3-gallate (EGCG), the most abundant and powerful cancer chemopreventive polyphenol in green tea [13], induces CTR1 expression and inhibits its rapid degradation by cDDP in ovarian cancer cells and mouse xenografts [14]. EGCG in combination with cDDP improves cDDP and DNA-platinum adduct accumulation, which enhances ovarian cancer cell sensitivity to the chemotherapeutic agent [14].

Hsa-mir-98-5p belongs to the let-7 family of microRNAs (miRNAs) [15–17] and is dysregulated in cancers of the lung [18], breast [19] and colon [20], and in esophageal squamous tumors [21]. Hsa-mir-98- 5p inhibits tumor cell growth and metastasis in oral squamous cell carcinoma by targeting IGFIR [22], and its overexpression prevents glioma cell line invasion by downregulating IKKε [23]. Hsa-mir-98-5p can also restrain stem cell proliferation in ovarian cancer [24] and inhibit prostate cancer growth [25]. In NSCLC, hsa-mir-98-5p can bind ITGB3 to suppress cancer proliferation, migration and invasion [26], and in breast cancer, up- regulation of the miRNA can serve as a biomarker [27]. Hsa-mir-98-5p reportedly inhibits tumor suppressor FUS1 expression in lung cancer [28]. Our previous study indicated that EGCG suppresses hsa-mir-98-5p expression by upregulating p53, and thus cDDP efficacy is enhanced in NSCLC cells [29]. Our bioinformatics analysis suggested that CTR1 is a putative target of hsa-mir-98-5p.

Non-coding RNAs (ncRNAs) are receiving increased attention due to their roles in post-transcriptional regulation and cell growth, differentiation and proliferation [30]. Long non-coding RNAs (lncRNAs) are polyadenylated ncRNAs of more than 200 nucleotides, and are found in the nucleus and cytosol [31]. LncRNAs regulate gene expression through epigenetic modification, mRNA splicing, genomic imprinting or control of transcription or translation [32]. Accumulating evidence has confirmed the interplay between miRNAs and lncRNAs, especially in carcinogenesis [33–36]. Recent studies have showed that lncRNAs can act as miRNA sponges, reducing their regulatory effects of miRNAs [33]. The lncRNA, nuclear enriched abundant transcript 1 (NEAT1), acts as an essential nuclear structural component [37]. NEAT1 dysregulation facilitates tumorigenesis in a variety of human cancers [38–41]. NEAT1 overexpression is associated with poor prognosis in breast and esophageal cancers [42–43], and with progression and metastasis in lung cancer. NEAT1 is reportedly upregulated in NSCLC patient plasma [44] and in NSCLC tissues in general as compared to adjacent normal lung tissues [39]. In lung cancer, NEAT1 is regulated by microRNA-449a, which can inhibit tumor cell growth [45].

In the current study, we explored the mechanism of EGCG-induced CTR1 in NSCLC *in vitro* and *in vivo*, and investigated whether microRNAs or lncRNAs were involved in CTR1 regulation. Bioinformatics analysis suggested that hsa-mir-98-5p has complementary binding with NEAT1. We hypothesized that NEAT1 and hsa-mir-98-5p could positively and negatively regulate EGCG-induced CTR1 gene expression, respectively. We explored the interaction between hsa-mir-98-5p, NEAT1 and CTR1 *in vitro* and *in vivo*. Novel mechanisms of EGCG-induced CTR1 and cDDP sensitivity enhancement in NSCLC are described.

## RESULTS

### EGCG sensitized NSCLC cells to cDDP

**Figure 1:**
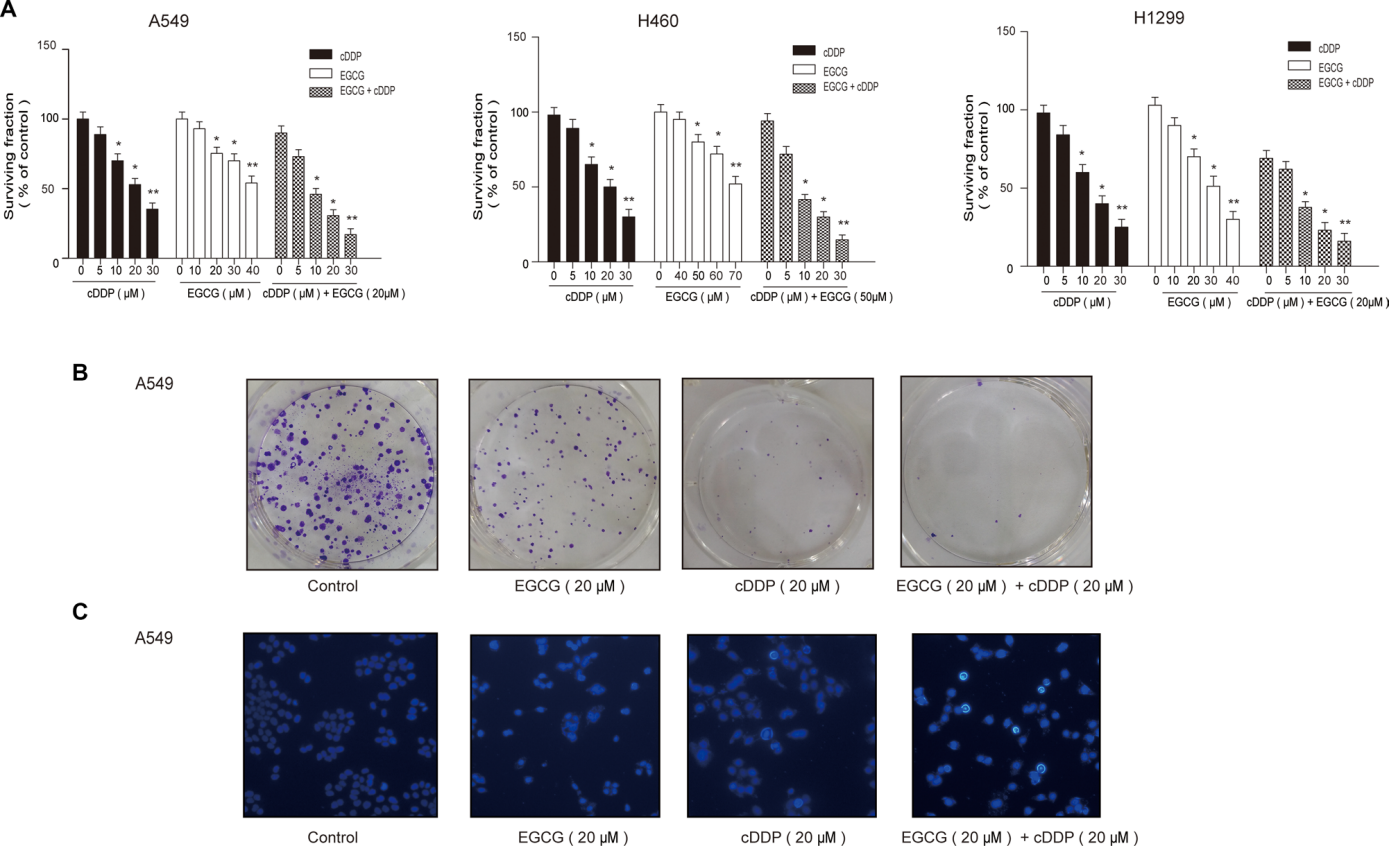
EGCG enhanced lung cancer cell sensitivity to cDDP (**A**) A549 cells were incubated with indicated concentrations of cDDP and EGCG alone or in combination for 24 h, and MTT assay was performed to assess cell survival. (**B**) Cells were treated with cDDP and EGCG alone or in combination for 48 h. Colonies were counted after two weeks. (**C**) Cell apoptosis was assessed by Hoechest staining. Error bars represent the mean ± SD of at least triplicate experiments. *P < 0.05, **P < 0.01.

To verify the effect of EGCG on cDDP sensitivity, NSCLC A459, H460 and H1299 cells were treated with varying concentrations of cDDP and EGCG, alone or in combination, for 24 h. MTT assays were performed to assess cell survival. cDDP or EGCG alone inhibited cell growth, and this effect was enhanced by treatment with both drugs in combination (Figure 1A). In A549 cells, the IC_50_ was 15.09 ± 0.25 μM (mean ± SEM) for cDDP alone and 8.21 ± 0.36 μM for the combined group. In H460 and H1299 cells, combination therapy decreased the IC_50_ by 41% and 47%, respectively. To exclude additive effects, “combination index” (CI) was used to assess EGCG-cDDP interaction. We observed that even at lower concentrations, the combination of 20 μM EGCG and 10 μM cDDP demonstrated synergistic anti-proliferation effects with CI values of 0.72, 0.78 and 0.65 in A549, H460 and H1299 cells, respectively.

Colony formation assays were used to investigate the effects of EGCG and cDDP on cell proliferation. A549 cells were treated with varying concentrations of cDDP and EGCG, alone or in combination, for 48 h. Both EGCG and cDDP inhibited colony formation and growth, but the inhibition was greatest with combined treatment (Figure 1B).

Hoechst 33258 staining was performed to detect treatment-induced apoptosis in A549 cells. EGCG and cDDP together increased apoptosis more than either treatment alone (Figure 1C).

### EGCG increased Pt and DNA-Pt adduct levels by inducing CTR1 expression

**Figure 2:**
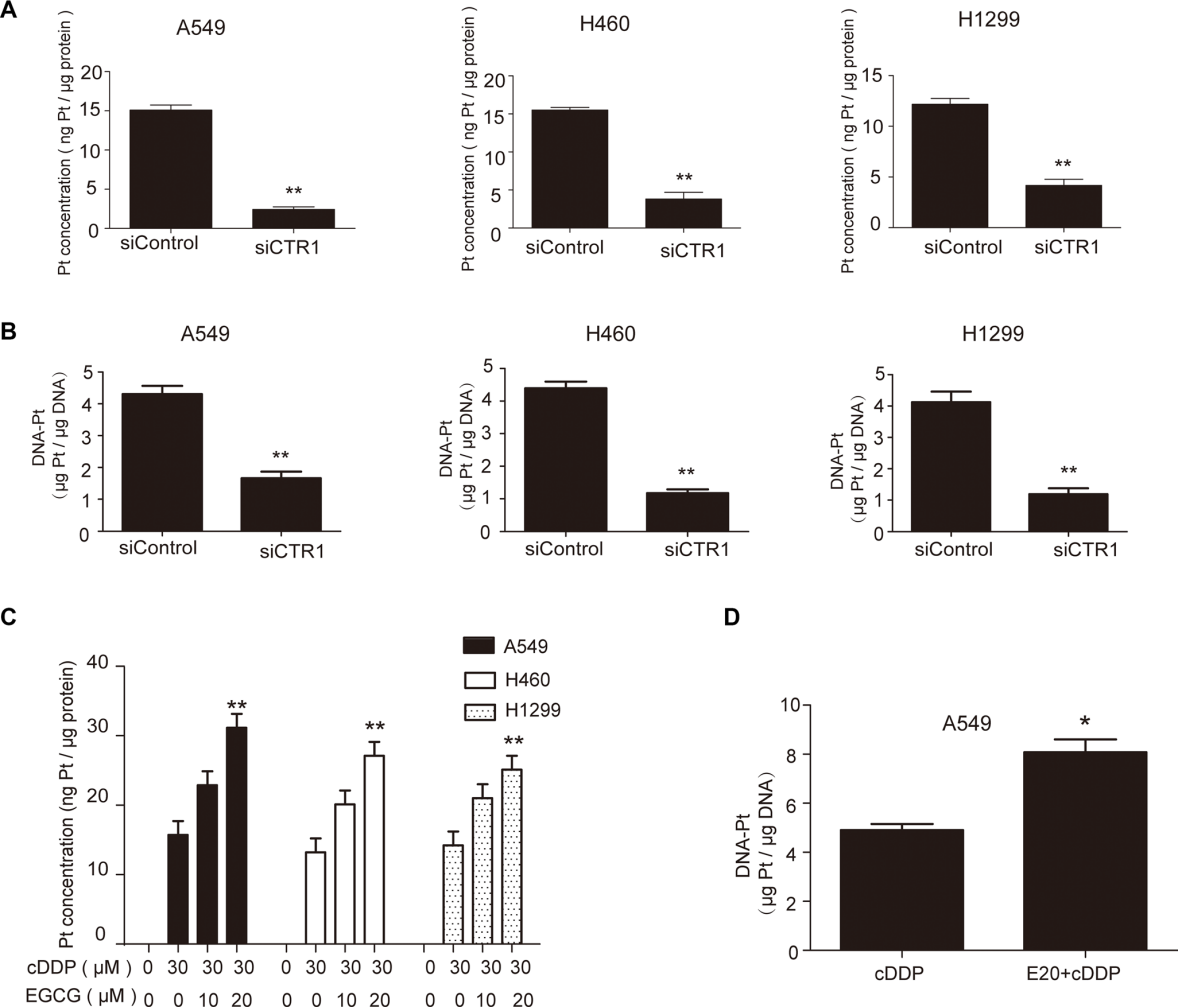
EGCG increased cDDP and DNA-Pt adduct accumulation in NSCLC cells (**A–B**) NSCLC cells were transfected with CTR1 or control siRNA and then incubated with 30 μM cDDP for 4 h. ICP-MS results showed that Pt A. and DNA-Pt adduct accumulation B. were reduced by CTR1 knockdown. (**C**) A549, H460 and H1299 cells were treated with various concentrations of EGCG for 24 h then incubated with 30 μM cDDP for 4 h. ICP-MS assay showed an EGCG-induced increase in Pt accumulation. (**D**) A549 cells were treated with 20 μM EGCG and then incubated with 30 μM cDDP for 4 h. Total DNA was extracted and ICP-MS assay showed an EGCG-induced increase in DNA-Pt adduct accumulation. Error bars represent the mean ± SD of at least triplicate experiments. *P < 0.05, **P < 0.01.

Since CTR1 is a major cDDP transporter, it is expected to regulate Pt and DNA-Pt adduct levels in tumor cells. CTR1 knockdown decreased intracellular Pt and DNA-Pt adduct accumulation in NSCLC cells (Figure 2A–2B). In addition, 20 μM EGCG promoted Pt accumulation and enhanced DNA-Pt adduct concentration in A549 cells (Figure 2C–2D).

**Figure 3:**
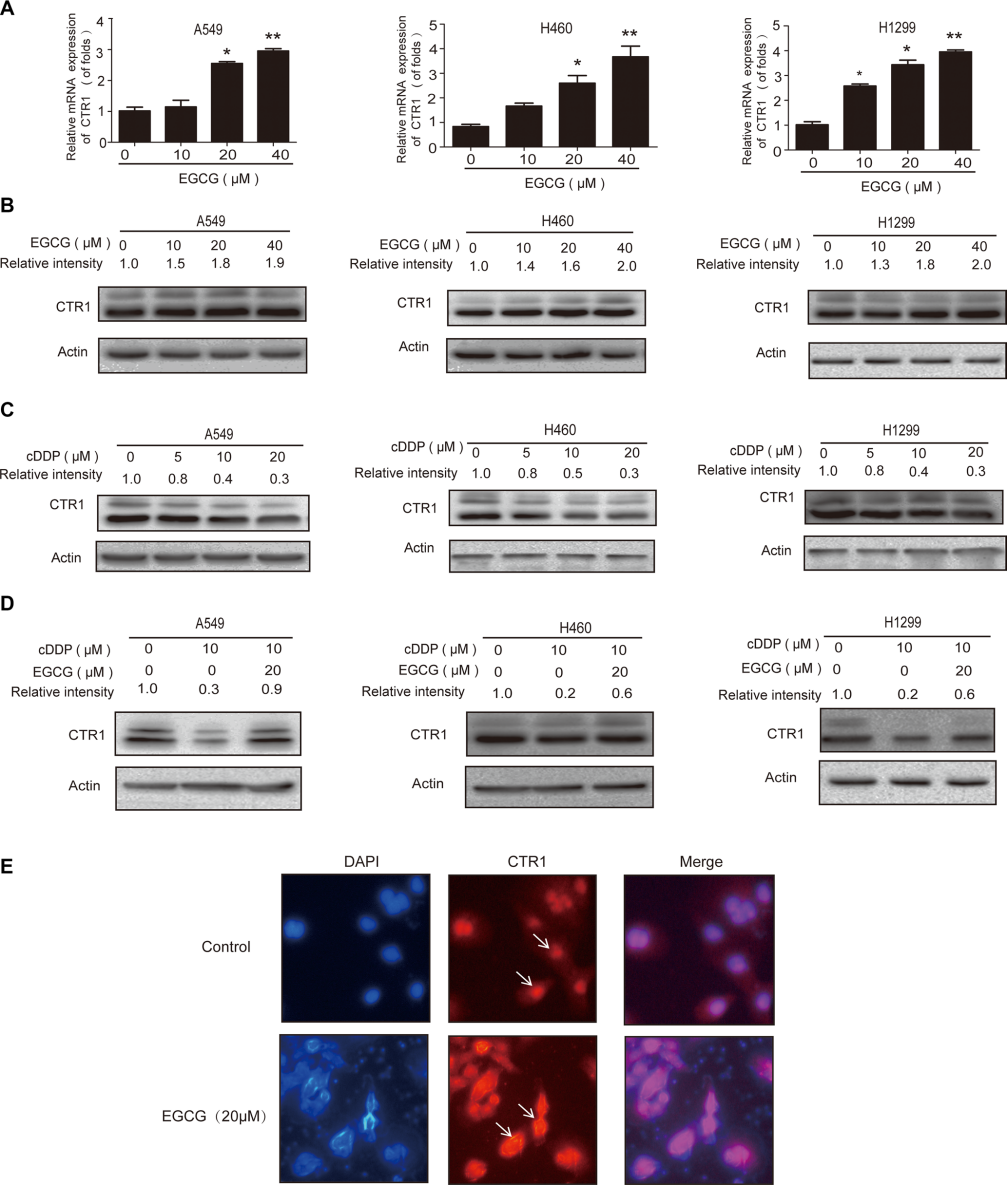
EGCG induced CTR1 expression and reversed cDDP-triggered CTR1 degradation (**A**) A549, H460 and H1299 cells were treated with the indicated doses of EGCG for 24 h. Real-time PCR was used to analyze CTR1 expression with GAPDH as an internal control. (**B–D**) CTR1 protein levels were assessed via western blotting with β-actin as a loading control. Effects of EGCG alone B. cDDP alone (C). or in combination (D) on CTR1 protein level, with β-actin as an internal control. (**E**) A549 cells were treated with the indicated doses of EGCG for 24 h. Immunofluorescence microscopy was performed to identify the localization of CTR1 proteins. Error bars represent the mean ± SD of at least triplicate experiments. *P < 0.05, **P < 0.01.

Real-time PCR was used to measure EGCG-induced CTR1 expression. CTR1 mRNA levels were elevated in a dose-dependent manner after EGCG treatment in A549, H460 and H1299 cells (Figure 3A). Western blot analysis showed that CTR1 protein levels were increased following EGCG treatment (Figure 3B). The molecular weight of CTR1 was included in Supplementary Figure S1.

Our previous study found that EGCG reversed cDDP-triggered CTR1 degradation in ovarian cancer cells [14], and the present study confirmed this effect in NSCLC cells (Figure 3C–3D). Taken together, these results suggest that EGCG-induced CTR1 expression increased cellular Pt levels.

Altered localization of transport proteins has an impact on their function. Copper transporters have to move to cell surface to perform metal transportation [46–47]. It is assumed that EGCG may also increase the level of CTR1 on cell surface. To investigate the localization of CTR1 proteins after EGCG treatment, immunofluorescence microscopy was performed. As shown in Figure 3E, CTR1 was located around the nucleus in A549 cells. However, when the cells were incubated with the indicated doses of EGCG, the localization of CTR1 proteins changed from peri-nucleus to cytoplasma (Figure 3E), which made it easier to transport cisplatin.

In summary, all these results exhibited that EGCG not only induced the expression of CTR1 but also affected CTR1 intracellular localization, which increased the functional CTR1.

### The hsa-mir-98-5p/NEAT1 axis regulates CTR1 in cDDP-sensitive NSCLC cells

**Figure 4:**
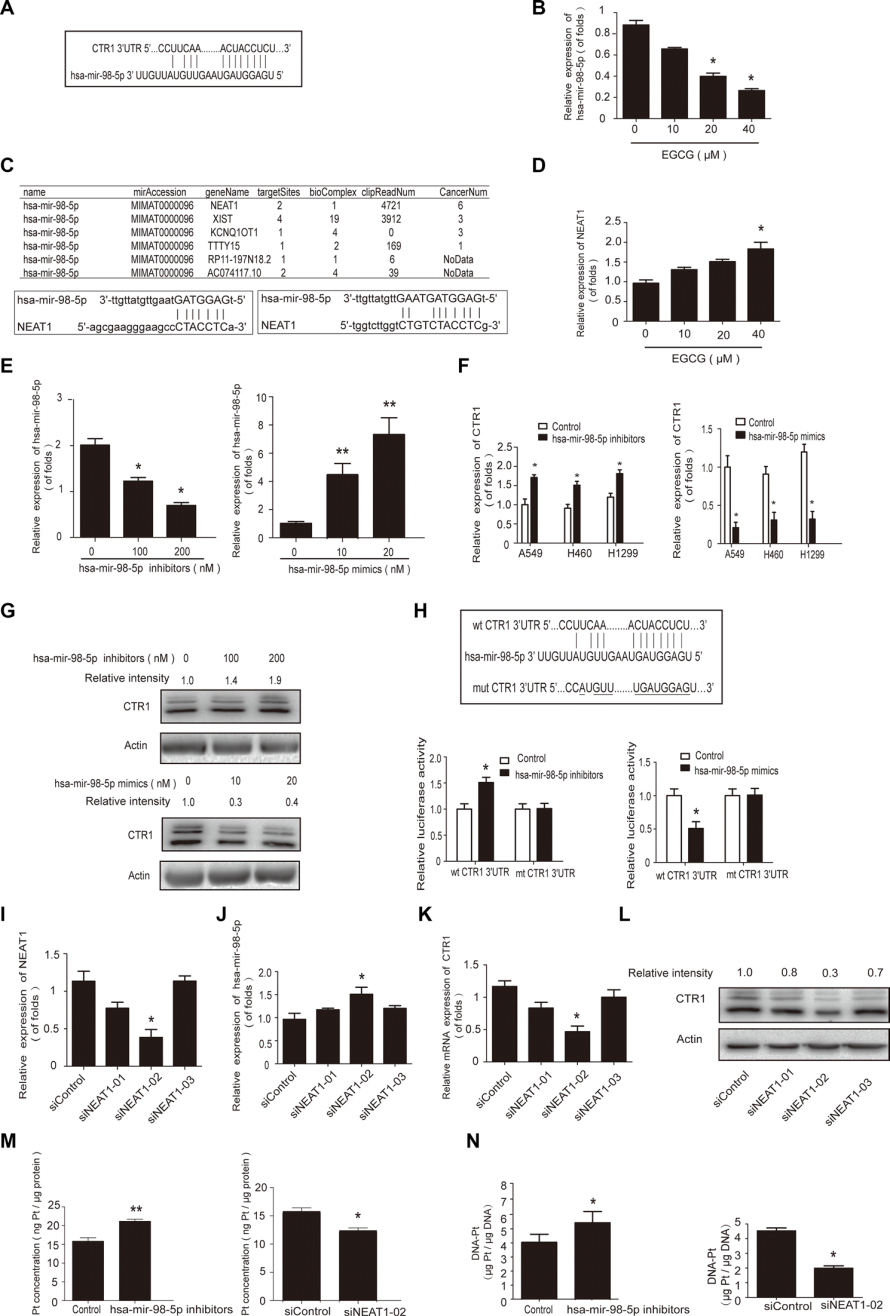
The hsa-mir-98-5p/NEAT1 axis regulated CTR1 in A549 cells (**A**) Binding between hsa-mir-98-5p and CTR1 was predicted via the TargetScan, Starbase, miRanda and miRDB databases. (**B**) A549 cells were incubated with the indicated doses of EGCG for 24 h. Real-time PCR was used to detect hsa-mir-98-5p, with U6 as an internal control. (**C**) Complementary biding sites between hsa-mir-98-5p and NEAT1 were predicted via ChipBase, LncRNAdb and StarBase. (**D**) A549 cells were incubated with the indicated doses of EGCG for 24 h. Real-time PCR was used to detect NEAT1 with GAPDH as a loading control. (**E**) A549 cells were transfected with hsa-mir-98-5p mimics, inhibitors or their parental negative control. (**F**) Real-time PCR was performed to measure the CTR1 levels after transfection, with GAPDH as an internal control. (**G**) Effect of hsa-mir-98-5p on CTR1 protein levels. Western blotting was conducted to measure CTR1 protein with β-actin as a loading control. (**H**) Hsa-mir-98-5p inhibitors or mimics were co-transfected with the wild type or mutated 3′UTR of CTR1 in A549 cells. Dual luciferase assays were performed to validate CTR1 is a direct target of hsa-mir-98-5p. (**I**) A549 cells were transfected with three NEAT1 siRNAs or siRNA control. SiRNA-02 showed the best knockdown effect. (**J–K**) Real-time PCR was used to detect hsa-mir-98-5p J. and CTR1 expression K. following NEAT1 knockdown. (**L**) Western blotting was performed to detect CTR1 protein level. (**M–N**) A549 cells were incubated with 30 μM cDDP for 4 h after hsa-mir-98-5p inhibition or NEAT1 knockdown. ICP-MS was used to measure Pt M. and DNA-Pt adduct accumulation N. Error bars represent the mean ± SD of at least triplicate experiments. *P < 0.05, **P < 0.01.

Our previous findings indicated that EGCG enhanced cDDP efficacy by inhibiting hsa-mir-98-5p in A549 cells [29], and we speculated that CTR1 could be regulated by microRNAs. Using the TargetScan, Starbase, miRanda and miRDB databases, we predicted CTR1 as a putative hsa-mir-98-5p target (Figure 4A). In agreement with our previous study, when A549 cells were treated with EGCG for 24 h, hsa-mir-98-5p expression was inhibited (Figure 4B).

Bioinformatics analysis, including LncRNAdb and StarBase were used to explore whether lncRNAs are involved in regulating hsa-mir-98-5p. NEAT1 was predicted to have complementary binding sites with hsa-mir-98-5p (Figure 4C). NEAT1 expression was upregulated in A549 cells treated with various concentrations of EGCG for 24 h (Figure 4D).

We hypothesized that NEAT1 and hsa-mir-98-5p were the potential positive and negative regulators of CTR1, respectively. To assess the relationship between hsa-mir-98- 5p, NEAT1 and CTR1, hsa-mir-98-5p mimics and inhibitors were transfected into A549 cells (Figure 4E). CTR1 expression was downregulated by hsa-mir-98-5p mimics and upregulated by hsa-mir-98-5p inhibitors (Figure 4F–4G).

To determine whether or not hsa-mir-98-5p directly targeted the CTR1 mRNA 3′UTR, A549 cells were transfected with wild-type or mutated CTR1 3′UTR and dual luciferase activity was analyzed (Figure 4H). Transfection with the wild-type 3′UTR and hsa-mir-98- 5p inhibitors elevated luciferase activity, while the mimics reduced luciferase activity. The results indicated that CTR1 was a direct target of hsa-mir-98-5p.

NEAT1 siRNA was used to investigate how NEAT1 regulated hsa-mir-98-5p and CTR1. Three pre-designed NEAT1 siRNAs or siRNA controls were transfected into A549 cells and NEAT1 siRNA-02 had the best knockdown effect (Figure 4I). NEAT1 knockdown increased hsa-mir-98- 5p expression (Figure 4J) and decreased CTR1 expression (Figure 4K–4L). Hsa-mir-98-5p inhibition significantly increased intracellular Pt and DNA-Pt adduct accumulation in A549 cells, while NEAT1 knockdown suppressed Pt and DNA-Pt adduct absorption (Figure 4M–4N). These results showed that the hsa-mir-98-5p/NEAT1 axis regulated CTR1 in cDDP-sensitive NSCLC cells.

### EGCG sensitized cDDP-resistant A549/cDDP cells to cDDP through NEAT1/hsa-mir-98-5p/CTR1

**Figure 5:**
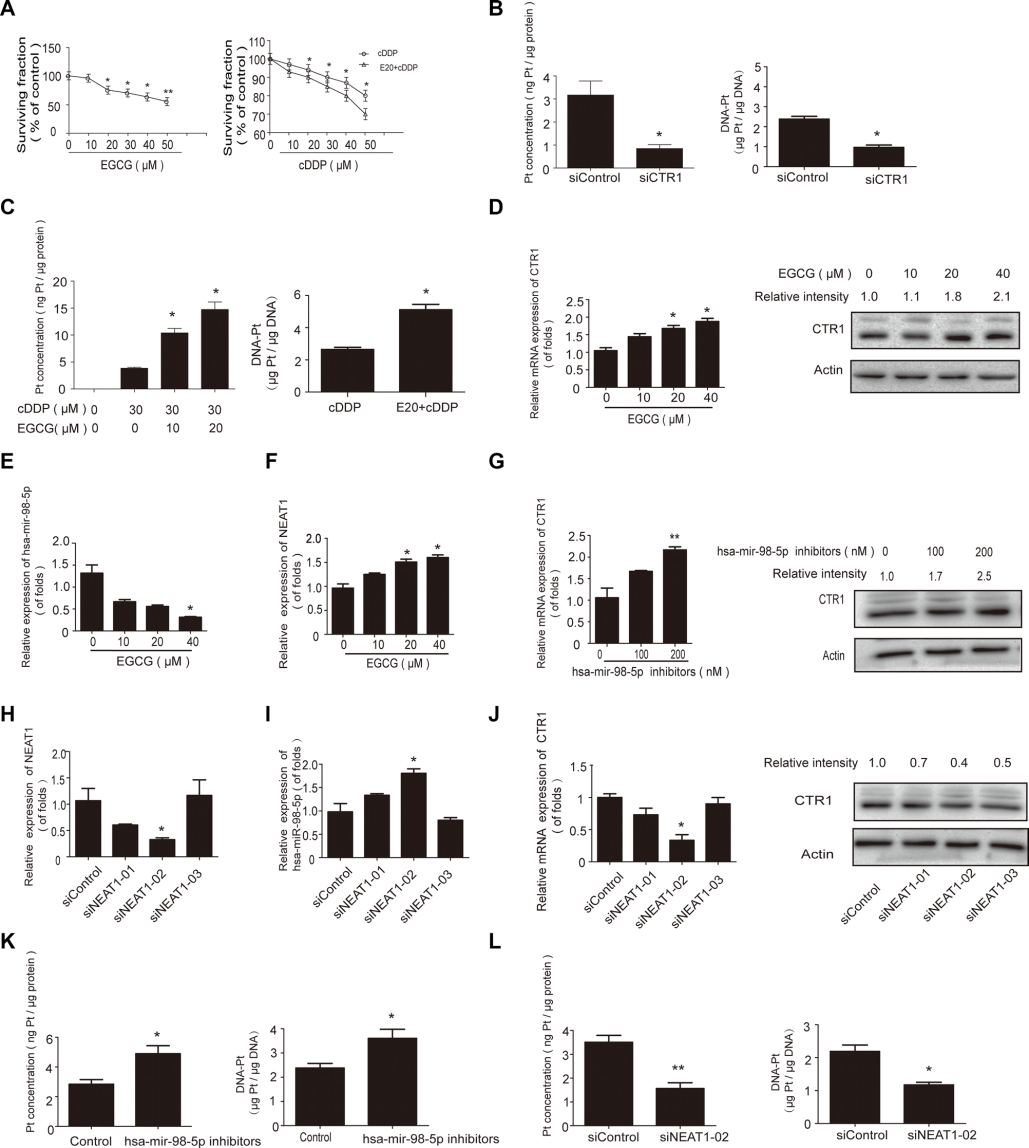
EGCG sensitized A549/cDDP (cDDP resistant) cells to cDDP via the NEAT1/hsa-mir-98-5p/CTR1 axis (**A**) A549/cDDP cells were treated with cDDP alone or in combination with EGCG for 24 h followed by MTT analysis. (**B**) ICP-MS was used to measure Pt and DNA-Pt adduct accumulation in A549/cDDP cells following CTR1 knockdown. (**C**) A549/cDDP cells were incubated with the indicated EGCG concentrations for 24 h and then treated with 30 μM cDDP for 4 h. ICP-MS was used to detect Pt and DNA-Pt adduct accumulation. (**D**) A549/cDDP cells were treated with the indicated doses of EGCG for 24 h. Real-time PCR was performed to analyze CTR1 expression and western blot analysis was carried out to analyse CTR1 protein level. (**E–F**) Real-time PCR was performed to detect has-mir-98-5p E. and NEAT1 F. after A549/cDDP cells were incubated with the indiacted concentrations of EGCG for 24 h. (**G**) A549/cDDP cells were transfected with hsa-mir-98-5p mimics, inhibitors or their parental negative control. Real-time PCR and western blotting were conducted to detect CTR1. (**H**) Real-time PCR was performed to measure the transfection effects of NEAT1 siRNA. (**I–J**) Effects of NEAT1 on hsa-mir-98-5p I. and CTR1 J. were measured by real-time PCR and western blotting. (**K–L**) Cells were incubated with 30 μM cDDP for 4 h after inhibition of hsa-mir-98-5p or NEAT1. ICP-MS assay was applied to measure Pt accumulation K. and DNA-Pt adducts L. Error bars represent the mean ± SD of at least triplicate experiments. *P < 0.05, **P < 0.01.

cDDP-resistant A549 cells were employed to assess whether or not EGCG could enhance cDDP sensitivity and whether NEAT1/hsa-mir-98-5p was involved in CTR1 regulation under conditions of cDDP insensitivity. Treatment with cDDP and EGCG together inhibited A549/cDDP cell growth (Figure 5A). In addition, CTR1 knockdown greatly inhibited Pt and DNA-Pt adduct accumulation in A549/cDDP cells (Figure 5B), whereas EGCG promoted Pt and DNA-Pt adduct absorption (Figure 5C). CTR1 and NEAT1 expression were elevated in A549/cDDP cells treated with EGCG (Figure 5D, 5F), whereas hsa-mir-98-5p expression was decreased (Figure 5E).

Hsa-mir-98-5p inhibitors increased CTR1 expression (Figure 5G). NEAT1 inhibited hsa-mir-98- 5p expression and increased CTR1 expression in A549/cDDP cells (Figure 5H–5J). Hsa-mir-98-5p inhibitors enhanced cDDP and DNA-Pt adduct absorption whereas NEAT1 knockdown decreased absorption (Figure 5K–5L). These results showed that NEAT1/hsa-mir-98-5p regulated EGCG-induced CTR1 in cDDP resistant cells.

### EGCG enhanced cDDP sensitivity in A549 cell nude mouse xenografts

**Figure 6:**
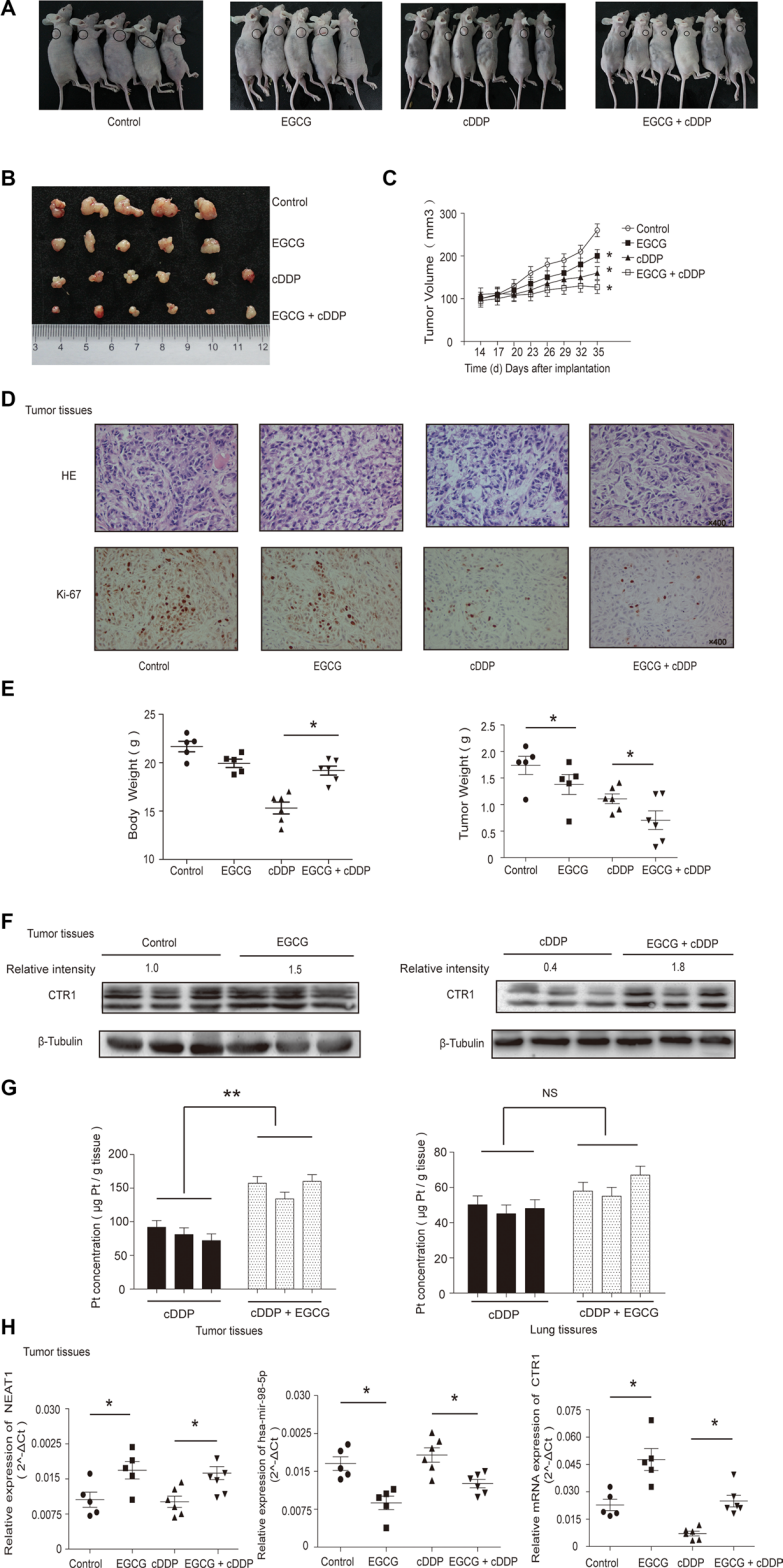
EGCG enhanced cDDP sensitivity in xenografted A549 cells (**A**) Twenty-two 4-5-week old female BALB/c nude mice were injected with 5 × 10^6^ A549 cells each. Four treatment groups included Control (five mice), EGCG (five mice), cDDP (six mice) and EGCG + cDDP (six mice). (**B**) Solid tumors were peeled from mouse subcutaneous tissue. (**C**) Tumor size changed in a time-dependent manner. (**D**) H&E and IHC staining of Ki-67 in tumor tissues. (**E**) Body and tumor weights were measured when mice were sacrificed. (**F**) Western blotting was used to assess CTR1 levels in tumor tissues, with β-tubulin as an internal control. (**G**) ICP-MS was used to assess Pt accumulation in tumor and lung tissues. (**H**) NEAT1, hsa-mir-98-5p and CTR1 in tumor tissues were detected by real-time PCR. Error bars represent the mean ± SD of at least triplicate experiments.*P < 0.05, **P < 0.01.

An A549 cell nude mouse xenograft model was established to determine whether or not EGCG promoted cDDP sensitivity *in vivo*. Mice were divided into four groups (Figure 6A) and tumors were peeled from nude mice subcutis (Figure 6B). In agreement with *in vitro* results, EGCG and cDDP independently inhibited tumor growth, and combination therapy enhanced this effect (Figure 6C). Immunohistochemistry (IHC) showed that combination therapy repressed Ki-67 (Figure 6D). EGCG treatment significantly reduced cDDP-induced weight loss in mice and the combination therapy most effectively inhibited tumor growth (Figure 6E).

EGCG prevented cDDP-induced CTR1 protein degradation, which was consistent with *in vitro* results (Figure 6F). Consequently, we observed that EGCG promoted Pt absorption in tumor tissues but not in lung tissues (Figure 6G). NEAT1, hsa-mir-98-5p and CTR1 mRNA extracted from tumor tissues was quantified by real-time PCR (Figure 6H). EGCG treatment upregulated NEAT1 and downregulated hsa-mir-98-5p compared to the control. The results showed that EGCG stimulated CTR1 expression, indicating that NEAT1 upregulated EGCG-induced CTR1 by sponging hsa-mir-98-5p *in vivo* (Figure 6H).

## DISCUSSION

**Figure 7:**
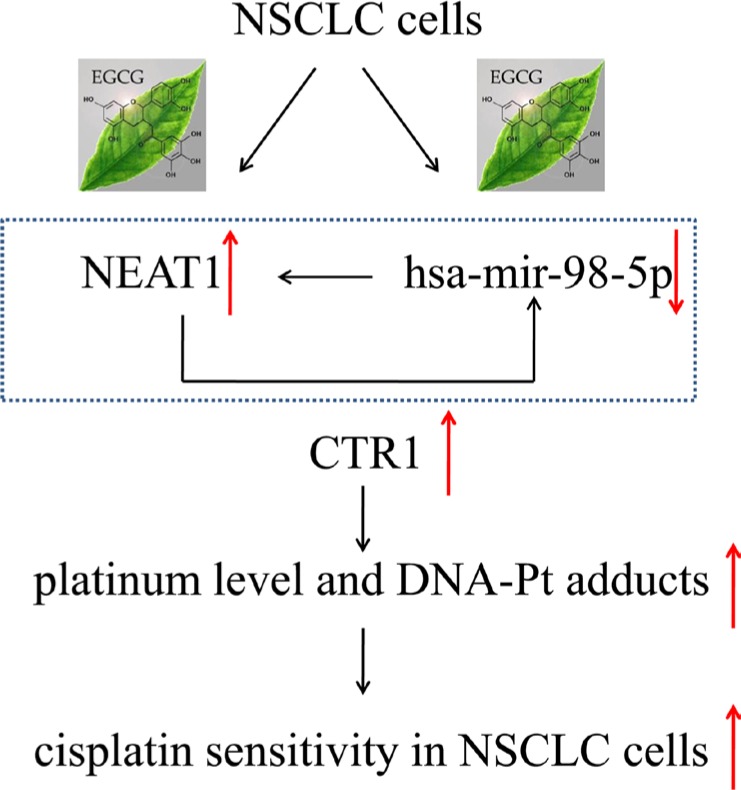
EGCG induced CTR1 and enhanced NSCLC cell sensitivity to cDDP via hsa-mir-98-5p and NEAT1 A schematic diagram of NEAT1/hsa-mir-98-5p/CTR1 axis regulated by EGCG in NSCLC cells.

Our previous study demonstrated that the green tea polyphenol, EGCG, induced cDDP transporter CTR1 expression and enhanced cDDP sensitivity in ovarian cancer [14]. In the current study, we confirmed these results in NSCLC *in vitro* and *in vivo* and further explored the mechanism of EGCG-mediated CTR1 expression. Our study identified two non-coding RNAs, hsa-mir-98- 5p and NEAT1, which modulated CTR1 expression. To our knowledge, this is the first report to show that EGCG-induced CTR1 is regulated by hsa-mir-98-5p and NEAT1 in NSCLC cells (Figure 7).

The role of copper uptake protein CTR1 in transporting Pt drugs has been elaborated in many studies [7]. Our previous study reported that CTR1 knockdown modified cDDP sensitivity in ovarian cancer cells [14]. In the current study, we verified that CTR1 knockdown inhibited Pt and DNA-Pt adduct accumulation in NSCLC cells, whereas EGCG treatment enhanced this accumulation. EGCG has been reported to inhibit cDDP-induced CTR1 degradation in ovarian cancer [14]. We observed that cDDP also caused rapid CTR1 degradation in NSCLC cells. However, EGCG combined with cDDP blocked this degradation significantly (Figure 3D). Ubiquitination and proteosomal degradation are reportedly involved in cDDP-triggered degradation [48–50]. Proteasome inhibitors such as bortezomib, actacystin or MG132 can block cDDP-induced CTR1 loss once cDDP is presented [48]. Our previous results verified that MG132 prevents cDDP-induced CTR1 degradation in ovarian cancer [14]. EGCG is an ubiquitin-proteasome inhibitor and enhances the effects of chemotherapeutics [51]. We hypothesized that the ubiquitin-proteasome pathway played an important role in cDDP-induced CTR1 degradation, but the pathway by which EGCG inhibits CTR1 degradation needs further investigation. In addition, there are many members in the cDDP transporter family and the effects of EGCG on these other transporters need further exploration.

According to several studies, copper-lowering agents can induce CTR1 expression, promote cDDP uptake and enhance sensitivity to cDDP [52–53]. High concentrations of copper and cDDP trigger CTR1 internalization [53]. Thus, copper and Pt drugs can repress each other's uptake in a dose-dependent manner. Understanding the mechanism of cDDP transportation by CTR1 and identification of CTR1 regulators are of great importance.

Interactions between lncRNAs and miRNAs are involved in a wide range of human carcinomas [54–55]. Increasing evidence has revealed that lncRNAs and miRNAs interact via post-transcriptional mechanisms [54]. miRNAs can trigger lncRNA decay and reduced stabilities [33]. In human cervical carcinoma, lincRNA-p21, which is activated by p53, may be regulated by miRNA let-7b [56]. A well-known lncRNA, HOTAIR, is reportedly inhibited by let-7b overexpression [57]. On the other hand, lncRNAs serve as miRNA sponges/decoys and generate miRNAs. Linc-MD1 can sponge miR-133 and miR-135 away from their target mRNAs, thus upregulating MAML1 and MEF2C, respectively [58]. One study has indicated that lncRNA H19 generates miR-675 in colorectal cancer [59]. In addition, lncRNAs and miRNAs can compete with each other for mRNA binding sites. For instance, lncRNA ncNRFR could repress let-7 functions by competing with let-7 for endogenous target mRNAs in the malignant transformation of colonic epithelial cells [60]. Thus, lncRNAs and miRNAs form a complex regulation network in a variety of cancers.

LncRNA NEAT1 dysregulation has been reported in various cancers such as malignant glioma, esophageal carcinoma, colorectal carcinoma and lung cancer [36–39]. However, the role of NEAT1 in lung cancer has not been explored. Our study showed that NEAT1 could function as a competing endogenous lncRNA in lung cancer, mediating CTR1 by sponging hsa-mir-98-5p. Hsa-mir-98-5p and NEAT1 appear to negatively and positively regulate CTR1 gene expression, respectively. Further studies are needed to elucidate the NEAT1/hsa-mir-98-5p/CTR1 regulation network and determine whether NEAT1 mediates CTR1 directly.

Recent studies have reported that ncRNAs play significant rules in cDDP resistance in lung cancer [61–63]. For instance, microRNA-26a is reported to cause cDDP resistance in NSCLC by inhibiting E2F1, diminishing Akt phosphorylation and down-regulating Bcl2 expression [61]. In lung cancer, miR-15b regulates cDDP resistance by targeting PEBP4 [62], while lncRNA MEG3 regulates resistance by controlling p53 and Bcl-xl [63]. Our findings in NSCLC cell lines and xenografts support the use of EGCG as an adjuvant to combat cDDP resistance. This is the first reporting of a possible mechanism for EGCG-mediated CTR1 induction via NEAT1/hsa-mir-98-5p crosstalk in NSCLC. The results provide potential targets for NSCLC chemotherapy.

## MATERIALS AND METHODS

### Cell culture and reagents

Human lung carcinoma A549 cells and the NSCLC lines H460 and H1299 were obtained from the Chinese Academy of Sciences Committee on Type Culture Collection Cell Bank (Shanghai, China). The cDDP-resistant A549 cell line (A549/cDDP) was a gift from the School of Basic Medical Science of Nanjing Medical University. H460 cells were cultured in Dulbecco's Modified Eagle Medium (DMEM, GIBCO, Carlsbad, CA, USA). A549/cDDP, A549, and H1299 cells were cultured in RPMI 1640 medium supplemented with 10% heat-inactivated fetal bovine serum (FBS, GIBCO, Carlsbad, CA, USA), 100 U/ml penicillin and 100 mg/ml streptomycin. 2 μg/ml DDP was added to media to sustain A549/cDDP cell growth after attachment. Cells were incubated with 5% CO2 at 37°C. EGCG and cDDP powders were purchased from Sigma (St. Louis, MO, USA).

### MTT assay

Cell viability was evaluated by MTT assay. Two thousand cells per well were seeded in a 96-well plate overnight. EGCG and cDDP alone or in combination were dissolved in 200 μl media. After 24 or 48 h of treatment, cells were incubated with 20 μl of 5 mg/ml MTT solution (Amresco, OH, USA) for 4 h. MTT formazan crystals were dissolved in 200 μl DMSO (Lingfeng, Shanghai, China) and absorbance was measured at 490 nm using a micro plate reader (Tecan, Mannedorf, Switzerland).

Median-effect analysis was used to evaluate synergistic drug combinations *in vitro* [64]. This method used the “combination index” (CI) to evaluate synergy between CDDP and EGCG in combination against A549, H460 and H1299 cells *in vitro*. Values of CI < 1, CI = 1, and CI > 1 represent synergy, additivity and antagonism, respectively.

### Colony formation assay

Five hundred cells per well were seeded in 6-well plates after the indicated treatments. Medium was changed every three days. After two weeks, visible colonies were fixed and stained with crystal violet (Beyotime, Shanghai, China).

### Hoechst staining

Cells were seeded and incubated for 24 h in 6-well plates, and then exposed to EGCG or cDDP alone or in combination for 48 h. Cells were fixed in 4% paraformaldehyde for 15 min, washed three times with PBS and stained with 500 μl of Hoechst 33258 (Beyotime, Shanghai, China) for 5 min. After three PBS washes, stained nuclei were observed under an inverted fluorescence microscope (Olympus, Tokyo, Japan).

### Western blot analysis

Western blotting results were quantified using Image J software. Proteins were harvested from A549, H460, H1299 and A549/cDDP cells. Cells lysed in RIPA buffer containing PMSF (protease and phosphatase inhibitors) were quantified via BCA protein assay. Proteins separated on 10% SDS-PAGE (Invitrogen) were transferred onto PVDF membranes (PolyVinylidene Fluoride). After blocking in 5% defatted milk, membranes were incubated with primary antibodies overnight at 4°C. Membranes were washed with TBST and incubated with Horse Radish Peroxidase (HRP)-conjugated secondary antibodies for 1 h at room temperature. Primary antibodies included: anti-CTR1 (1:1000, Abcam, Cambridge, Britain), anti-β-actin (1:1000, BOSTER, Wuhan, China) and anti-β-tublin (1:1000, BOSTER, Wuhan, China). Secondary antibodies included: HRP-Conjugated AffiniPure Goat Anti-Rabbit IgG (1:2000, ZSGB-BIO, Beijing, China) and HRP-Conjugated AffiniPure Goat Anti-Mouse IgG (1:2000, ZSGB-BIO, Beijing, China). Chemiluminesence western blotting reagents (Cell Signaling Technology, Danvers, MA, USA) were used to detect immunoreactive proteins. Protein bands were measured using Eagle Eye II software.

### Immunofluorescence microscopy

For immunofluorescence microscopy, cells were seeded and grown on 6-well plates. After the indicated treatments for 24 h, cells were washed with PBS and fixed with 4% paraformaldehyde for 15 min. The cells were washed three times with PBST and blocked for 60 min with 2% BSA at room temperature. Then, the cells were probed with CTR1 antibody (Abcam, Cambridge, Britain) diluted 1:100 in 2% BSA overnight at 4°C. After antibody binding, the samples were washed three times with PBST and incubated with the secondary antibodies DyLight549 Anti-Rabbit IgG (Jackson ImmunoResearch, PA, USA) diluted 1:100 in 2% BSA. DNA specific fluorochrome 4, 6-diamidino-2-phenylindol (DAPI) (Beyotime, Shanghai, China) was used to stain the cell nucleus. The stained cells were observed with confocal laser microscope fluorescence (Olympus, Tokyo, Japan).

### Bioinformatics analysis

TargetScan (http://www.targetscan.org/), Starbase (http://starbase.sysu.edu.cn/), miRanda (http://www.microrna.org/) and miRDB (http://www.mirdb.org/) databases were used to predict putative CTR1 microRNA targets. LncRNAdb (http://www.lncrnadb.org/) and StarBase (http://starbase.sysu.edu.cn/) were performed to identify specific lncRNAs regulated by hsa-mir-98-5p.

### RNA extraction, reverse transcription and real-time RT-PCR

Total RNA, miRNA and lncRNA were extracted using RNAiso Plus (TaKaRaBio Technology, Dalian, China). RNA was reverse transcribed using the Prime Script TM RT Master Mix (TaKaRa Bio Technology, Dalian, China) and qPCR was performed using SYBR Premix Ex Taq II (TaKaRaBio Technology, Dalian, China). qRT-PCR primers were provided in Supplementary Table S1. Hsa-mir-98-5p primers were obtained from RiboBio (Guangzhou, China). Human U6 RNA was used as an internal microRNA control. GAPDH was used as an internal mRNA and lncRNA control. Real-time PCR was performed using the Applied Biosystems 7300 Real Time PCR System (Applied Biosystems, Foster City, CA, USA). Expression was defined through the threshold cycle and fold change was calculated using the equation 2^−ΔΔCt^. Relative NEAT1, hsa-mir-98-5p and CTR1 mRNA levels in tumor tissues were expressed as 2^−ΔCt^.

### siRNA and microRNA transfection

Human CTR1 or control (RiboBio, Guangzhou, China) siRNAs were transfected into A549 and A549/cDDP cells with Lipofectamine 2000 (Invitrogen, Carlsbad, CA, USA) following the manufacturer's instructions. NEAT1 or control siRNAs were transfected into A549 or A549/cDDP cells with Lipofectamine 2000. Hsa-mir-98-5p mimics, inhibitors and their parental negative control was transfected into NSCLC A549, H460, H1299 cells and cDDP-resistant A549 cells.

### Luciferase activity assay

The wild-type (wt) and mutant (mut) hsa-mir-98-5p binding site in the 3′-UTR of CTR1 were synthesized and subcloned into the pGL3 Basic vector (Promega). 1 × 10^5^ A549 cells were seeded into 24-well plates for 24 h. Hsa-mir-98-5p mimics or inhibitors (RiboBio, Guangzhou, China) were cotransfected with 10 μg pLUC-wt-CTR1 or pLUC-mut-CTR1 using Lipofectamine 2000 (Invitrogen, Carlsbad, CA, USA). Luciferase activity was measured by the Dual-Luciferase Reporter Assay System (Promega, Madison, WI). Renilla luciferase activity was normalized to firefly luciferase activity.

### Ethics statement

This study was performed in strict accordance with the requirements in the Guide for the Care and Use of Laboratory Animals of the National Institutes of Health. The protocol was approved by the Committee on the Ethics of Animal Experiments of Nanjing medical university.

### Nude mouse xenograft studies

Twenty-two mice (BALB/c, nude, female, aged 4–5 weeks, weighed 16–18 g, purchased from Shanghai Animal Laboratory Center) were maintained in the Experimental Animal Center at Nanjing Medical University with appropriate sterile filter-capped cages. The lights were turned on at 7:00 am and turned off at 5:00 pm in the center, with 22 ± 1°C temperature and 55 ± 5% humidity. Wood shavings, feedstuffs and water were well provided in all cages.

We observed all mice every day to detect any abnormal behavior, such as weight loss, irritation, inability to drink, eat or jump, or inactivity when touched. Mice were injected subcutaneously in the front dorsum with exponentially-growing A549 cells (5 × 10^6^ each). Tumor lengths and widths were measured using calipers, and volumes were calculated using the following formula: volume (mm^3^) = length × width × width/2.

At two weeks post-transplantation, average tumor volume was about 50 mm^3^. A549 xenografts were randomized into four groups (five control mice, five mice in the EGCG group, six in the cDDP group and six in the combination group). Treatment methods were as follows: control (normal saline, 0.1 ml/10 g), EGCG (20 mg/kg), cDDP (5 mg/kg), and EGCG (20 mg/kg) with cDDP (5 mg/kg). Drugs were given every three days through intra-peritoneal injection. Body weights and tumor sizes were recorded three times a week. After two weeks of treatment, all mice were euthanized by cervical dislocation and tumor and lung tissues were isolated.

### Platinum (Pt) accumulation and Pt-DNA adducts in cells and animal tissues

Inductively coupled plasma mass spectrometry (ICP-MS) was employed to measure whole-cell Pt content [65]. For Pt measurement in cells, cells were digested in 65% nitric acid and protein concentrations were measured and normalized. Cell samples were diluted appropriately before ICP-MS analysis. To measure Pt in DNA, DNA was extracted using DNAzol (Invitrogen, Carlsbad, CA, USA). For normalization, DNA concentration was measured using a Nanodrop 2000 spectrophotometer (Thermo Scientific, Wilmington, DE, USA). DNA samples were digested in 5% nitric acid before ICP-MS analysis.

Tissue Pt accumulation was also detected by ICP-MS as previously described [66]. 0.1 g tissue samples from nude mice were dried overnight in a clean oven at 65°C. Dried tissues were weighed and pre-digested with 3 ml of 65% HNO_3_ overnight in screw-capped digestion jars. 1 ml 30% hydrogen peroxide (GFS Chemical, Powell, OH) was added before high-pressure digestion was conducted. After complete digestion, fluid was transferred from digestion jars and digested samples were diluted suitably. After vortexing, tissue samples were analyzed for Pt accumulation by ICP-MS.

### Hematoxylin and eosin and immunohistochemistry staining

Hematoxylin and eosin (H&E) and IHC staining were performed by the Department of Pathology, Affiliated Nanjing First Hospital of Nanjing Medical University. Image-Pro Plus software (Version 6.0, Media Cybernetics, Bethesda, MD, USA) was used to analyze staining results.

### Statistical analysis

All data were presented as the mean ± standard deviation (SD) of at least three independent experiments. Comparisons between quantitative variables were assessed using the student's *t* test and one-way ANOVA. Data were considered statistically significant when *P* < 0.05. SPSS 17.0 (SPSS Inc, Chicago, IL, USA) and GraphPad Prism v5.0 (Graphpad Software Inc) software was used for statistical analysis.

## SUPPLEMENTARY FIGURES AND TABLES


