# Supplementary material for: NEAT1 upregulates EGCG-induced CTR1 to enhance cisplatin sensitivity in lung cancer cells
Source: Oncotarget. 2016 May 30;7(28):43337–51. doi: 10.18632/oncotarget.9712 (PMC5190027; doi:10.18632/oncotarget.9712)
Supplement: Supplementary file 1 [file oncotarget-07-43337-s001.pdf]

NEAT1 upregulates EGCG-induced CTR1 to enhance cisplatin sensitivity in lung cancer cells

Supplementary Materials

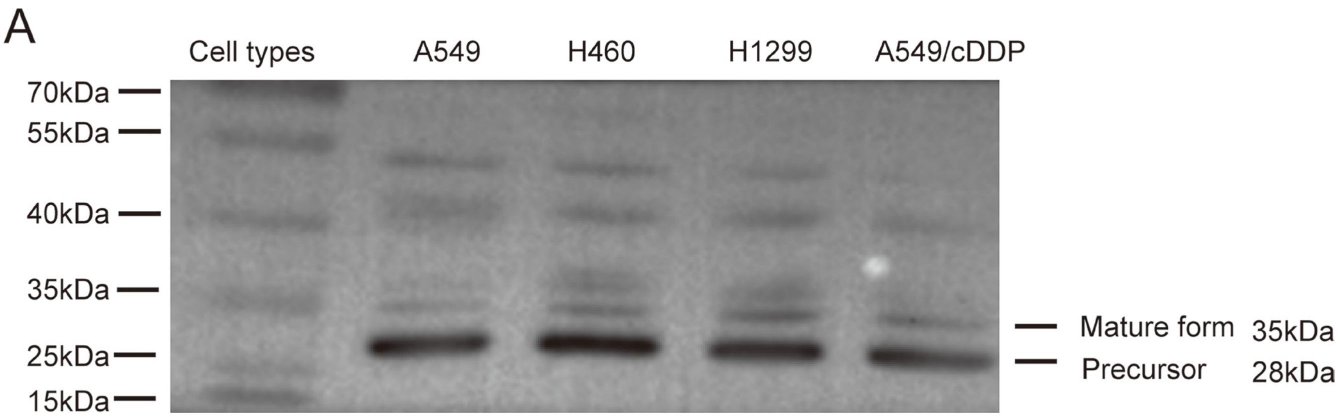

Supplementary Figure S1: Western blot bands of CTR1 in A549, H460, H1299 and A549/cDDP cells. (A) Proteins were harvested from A549, H460, H1299 and A549/cDDP cells. Western blotting was conducted to measure CTR1 proteins. Molecular weight of CTR1 was provided.

Supplementary Table S1: Primers used in quantitative real time-PCR

Name	Sequence
CTR1 forward primer	5′-GGGGATGAGCTATATGGACTCC-3′
CTR1 reveres primer	5′-TCACCAAACCGGAAAACAGTAG-3′
NEAT1 forward primer	5′-TGGCTAGCTCAGGGCTTCAG-3′
NEAT1 reveres primer	5′-TCTCCTTGCCAAGCTTCCTT-3′
GAPDH forward primer	5′-CAAGGTCATCCATGACAACTTTG-3′
GAPDH reveres primer	5′-GTCCACCACCCTGTTGCTGTAG-3′
